# Application of an AI-Based Pediatric Early Warning Score in the Pediatric Emergency Department: Cross-Sectional Study

**DOI:** 10.2196/89306

**Published:** 2026-05-19

**Authors:** Wanhua Xie, Xuan Shi, Meiqing Peng

**Affiliations:** 1 Outpatient Department, Guangzhou Women and Children's Medical Center, Guangzhou Medical University, 9 Jinsui Road, Guangzhou, Guangdong, 510623, China, 86 13725370379, 86 2038076020; 2 Pediatric Emergency Department, Guangzhou Women and Children's Medical Center, Guangzhou Medical University, Guangzhou, China

**Keywords:** pediatric early warning, artificial intelligence, AI, pediatric emergency, length of hospital stay, hospitalization costs

## Abstract

**Background:**

Pediatric emergency departments see a high volume of patients. Given that children often cannot describe their condition and there is a shortage of nursing staff, it is essential to identify the early warning signs of adverse conditions among children as quickly as possible. Current targeted care needs to be improved.

**Objective:**

This study aimed to investigate the application of an artificial intelligence (AI)–based version of the Pediatric Early Warning Score (PEWS) in a pediatric emergency observation unit, analyze the relationship between PEWS and disease severity, and assess its impact on the length of hospital stay and hospitalization costs after admission, thereby providing a reference for targeted nursing care.

**Methods:**

We performed a retrospective study. A total of 1233 pediatric patients admitted via the pediatric emergency department of a tertiary specialty hospital in Guangzhou from September 2023 to March 2024 were included. The patients were divided according to whether they triggered a PEWS early warning into an early warning group (PEWS ≥1) and a non–early warning group (PEWS=0) during emergency observation. Length of stay and hospitalization costs were compared between the early warning group and the non–early warning group. Differences between groups were assessed using the Mann-Whitney *U* test. We performed multivariable logistic regression to discuss the association of resource use metrics and PEWS status, adjusted by age, sex, and disease category (respiratory, neurological, and hematologic).

**Results:**

Of 1233 patients, 597 (48.4%) triggered the PEWS early warning (mean score 2.44, SD 1.41), and 636 (51.6%) did not. In the early warning group, 68 children were transferred to the intensive care unit, with a mean PEWS of 3.32 (SD 1.73). Compared with the non–early warning group, the early warning group had a longer hospital stay (*z*=−5.180; *P*<.001) and higher hospitalization costs (*z*=−6.500; *P*<.001), and the differences between groups were statistically significant (*P*<.001). Among the top 3 admission categories—respiratory, neurological, and hematologic diseases—children in the early warning group had significantly longer hospital stays and higher hospitalization costs (all *P*<.01). The β coefficient for length of hospital stay was 0.053 (SE 0.010; Wald *χ*²_1_=5.533; odds ratio 1.055, 95% CI 1.035‐1.075), while the β coefficient for hospitalization costs was 0.001 (SE 0.000; Wald *χ*²_1_=6.075; odds ratio 1.001, 95% CI 1.001‐1.001).

**Conclusions:**

Compared with the non–early warning group, the early warning group had significantly longer hospital stays and higher hospitalization costs (*P*<.001); similar patterns were observed within respiratory, neurological, and hematologic disease categories (all *P*<.01). These findings show differences between children who triggered the warning and children who did not, providing a reference for identifying critically ill children for targeted care.

## Introduction

### Background

Early identification of clinical deterioration in pediatric patients is critically important. The literature shows that children account for 8.5% to 14.0% of in-hospital sudden cardiopulmonary arrests, with a survival rate of only 15% to 33%; among survivors, the incidence of neurological impairment can be as high as 35% [1,2]. Analyses of the causes of pediatric cardiopulmonary arrest indicate that 61% are due to respiratory failure and 29% are caused by shock; most cases can be prevented or reversed with early recognition and intervention [3,4]. Another study from the United Kingdom also suggested that 26% to 43% of deaths among pediatric patients were avoidable or potentially avoidable [5]. In most cases, clinically detectable physiological changes—such as dyspnea and tachypnea, tachycardia, and altered mental or behavioral status—appear 6 to 8 hours before abrupt deterioration and cardiopulmonary arrest [6]. However, in pediatric emergency departments, children are often unable to describe their symptoms, nursing staff are relatively limited, and vigilance for early warning signs of deterioration may be insufficient. As a result, physiological changes may be overlooked, delaying early recognition [7]. Delays in early identification and intervention are closely associated with pediatric mortality, whereas timely and effective assessment, monitoring, and subsequent intervention and treatment can improve clinical outcomes [8-11]. Ruirui et al [12] investigated the impact of the Pediatric Early Warning Score (PEWS) on mortality in critically ill children with hand, foot, and mouth disease. Yejie and Qinghong [13] proposed the development of national or regional standardized PEWS criteria. Jie and Yan [14] noted that traditional nursing approaches often lack targeted care strategies tailored to different stages of disease progression.

### Objectives

This study aimed to evaluate the application of an artificial intelligence (AI)–based PEWS in the pediatric emergency observation unit. Specifically, we compared differences in the length of hospital stay, hospitalization costs, and other outcomes between a PEWS early warning group and a non–early warning group among pediatric patients with the top 3 specialty disease categories admitted via the pediatric emergency department. The study further explored the effectiveness of PEWS in the pediatric emergency setting and examined the relationship between PEWS, disease severity, and the next steps in patient care in the observation unit. By enabling timely and accurate identification of critically ill and potentially critically ill children, the approach may support prioritized medical and nursing care and provide references for targeted nursing care.

## Methods

### Study Design

This study used a cross-sectional design.

### General Information

A retrospective analysis was conducted of 1233 pediatric patients admitted via the pediatric emergency department of Guangzhou Women and Children’s Medical Center, Guangzhou Medical University, between September 2023 and March 2024. The included cases belonged to the top 3 admission categories: respiratory, neurological, and hematologic system diseases. Among them, n=597, 48.4%) patients were assigned to the early warning group and 51.6% (n=636) to the non–early warning group. Baseline characteristics were comparable between the 2 groups, with no statistically significant differences (all *P*>.05), as shown in Table 1.

**Table 1.** Comparison of baseline characteristics between the Pediatric Early Warning Score early warning group and the non–early warning group.

Group	Early warning group (n=597)	Non–early warning group (n=636)	Test statistic	*P* value
Sex, n (%)			0.5 (1)^a^	.49
Male	359 (60.1)	370 (58.2)		
Female	238 (39.9)	266 (41.8)		
Age (months), median (IQR)	33 (8-60)	30.5 (7.25-60)	−0.5^b^	.61
Admission categories, n (%)			0.8 (2)^a^	.68
Respiratory diseases	463 (77.6)	481 (75.6)		
Neurological diseases	75 (12.6)	90 (14.2)		
Hematologic diseases	59 (9.9)	65 (10.2)		

a *χ*2 (*df*).

b *z* score.

### Calculation of Sample Size

This study adopted a retrospective design with a fixed time window, and the sample size was naturally determined by case availability during the study period. From September 2023 to March 2024, all children who met the inclusion and exclusion criteria were consecutively enrolled, yielding a final sample of 1233 cases. Participants were stratified according to whether the PEWS system was triggered at the index emergency visit (PEWS ≥1), forming an early warning group (n=597, 48.4%) and a non–early warning group (n=636, 51.6%), which reflects the real-world distribution of emergency visits.

The inclusion criteria were (1) children admitted to hospital via the pediatric emergency department and (2) primary disease category ranked among the top 3 (eg, respiratory, neurological, or hematologic system diseases).

The exclusion criteria were (1) children with missing data and (2) children who discontinued treatment and were discharged after admission.

### Ethical Considerations

This study was approved by the Scientific Research Ethics Committee of Guangzhou Women and Children’s Medical Center (SFE-KL-35900). Because this study used only historical electronic medical records and information system data generated during the children’s previous medical treatment, it did not involve additional interventions or sample collection, and the research risks were no greater than the minimum risk. The ethics committee approved the exemption of informed consent from the parents of the children. During the study, patient-related information was anonymized, and the privacy and data security of the children were strictly protected.

### Study Instrument

The Brighton PEWS was used as the measurement instrument [15,16] (Table 2).

**Table 2.** Pediatric Early Warning Score (PEWS) criteria.

Parameter	0 point	1 point	2 points	3 points
Conscious behavior	Normal activity	Irritable	Irritable	Lethargic, comatose, or decreased response to pain
Cardiovascular	Pink skin color and CRT^a^ 1‐2 s	Pale skin color and CRT 3 s	Gray skin color, CRT 4 s, and heart rate increased by 20 beats/min above normal	Gray, cool, and clammy skin; CRT ≥5 s; and heart rate increased by 30 beats/min above normal or bradycardia
Respiration	Normal rate and no chest retractions	Respiratory rate 10 breaths/min above normal, FiO_2_^b^ >30%, and oxygen flow >3L/min	Respiratory rate 20 breaths/min above normal, obvious chest retractions, and FiO_2_ >40% or oxygen flow >6 L/min,	Respiratory rate 5 breaths/min below normal, chest retractions and grunting, and FiO_2_ >50% or oxygen flow >8 L/min

a CRT: capillary refill time.

b FiO_2_: fraction of inspired oxygen.

### Method of Use

Children for whom the early warning scoring system was triggered (PEWS ≥1) were assigned to the early warning group [17]; those without triggering of the system and with a PEWS of 0 were assigned to the non–early warning group.

Pediatric emergency nurses measured and monitored patients’ vital signs and mental status, recording data in the information system. When one or more indicators reached abnormal thresholds, the system automatically activated the PEWS form. Nurses then completed additional evaluations for other indicators. The system calculated the PEWS according to predefined rules, displaying it on both the PEWS form and nursing records with corresponding warning alerts and color indicators. Nurses took appropriate actions based on the score (Table 3).

For traditional PEWS, pediatric emergency nurses measured and monitored patients’ vital signs and mental status, recording data in the information system. When one or more indicators reached abnormal thresholds, the traditional PEWS system did not activate the PEWS form or display warning alerts or color indicators on the PEWS form or nursing records.

The PEWS system uses different warning colors: a total PEWS of 0 to 1 is a green alert, 2 is a yellow alert, and 3 is an orange alert. A red alert is triggered when the total score reaches 4 or above, when the score increases by more than 2 points, or when any single indicator reaches 3 points. In such cases, the system activates a critical-value mode and displays warnings in the physician’s electronic medical record.

The PEWS system used a deep learning model to automatically calculate scores and trigger alerts. For structured tabular data, ensemble models such as gradient boosting decision trees and random forests can provide feature importance rankings, which enhances the interpretability of the system. Meanwhile, to make fuller use of pediatric patients’ time-series vital sign data, recurrent neural networks were applied to model temporal dependencies and dynamic trends.

**Table 3.** Handling guidelines*.*

Score	Intervening measure
0 to 1 points	No treatment is required Continue with observation
2 points	Notify the senior nurse Consider whether there is pain or fever and calculate fluid balance and urine output Conduct condition observation according to the specific specialty and continue electrocardiogram monitoring if necessary
3 points	Observe the patient according to the situation and continuously monitor the patient Notify the nursing team lead or head nurse After the nursing team lead or head nurse conducts an assessment, notify the on-duty physician based on the patient’s condition
4 points or an increase of more than 2 points in the score	Notify the on-duty physician to report to the site within 15 minutes Conduct close monitoring of the child in accordance with their specific condition and maintain the use of monitoring equipment Initiate treatment for the patient as promptly as possible based on their clinical condition
≥4 points or any single indicator reaching 3 points	Inform the on-duty physician to rush to the scene immediately Transfer to the intensive care unit, prepare the emergency cart, and consider providing treatment or resuscitation to the patient as soon as possible based on the patient’s condition Depending on the patient’s condition, notify the senior physician and the physicians in the monitoring room or issue a blue alert

Cross-validation and other techniques were adopted during model training to prevent overfitting, and regularization was used to improve generalization ability. Finally, the trained model predicted a continuous PEWS based on the input real-time feature probabilities. The system automatically triggered different color warnings accordingly, providing clear, objective, and data-driven support for nurses’ triage decisions and interventions.

The PEWS system in this study was developed and validated based on historical electronic medical record data. The actual model architecture comprised three core components: (1) a deep learning model serving as the backbone network, responsible for automatically calculating the PEWS and triggering alerts; (2) ensemble learning models—including gradient boosting decision trees and random forests—used to process structured tabular data and provide feature importance rankings, thereby enhancing the system’s interpretability; and (3) a recurrent neural network specifically designed to model the dynamic trends and temporal dependencies of time-series vital signs data from pediatric patients.

Input data for the system included vital signs such as heart rate, respiratory rate, capillary refill time, skin color, level of consciousness and behavioral state (normal activity, irritability, lethargy, or coma), oxygen flow rate, and fraction of inspired oxygen (FiO₂). These measurements were recorded by pediatric emergency nurses into the hospital information system.

Model training used 5-fold cross-validation for data partitioning, with an 8:2 split between training and validation sets, and used L2 regularization to prevent overfitting. Ground-truth labels were defined based on historical PEWS records and final clinical outcomes, including intensive care unit (ICU) admission status, the length of hospital stay, and associated costs.

Model validation was performed using receiver operating characteristic (ROC) curves, calibration plots, and the Hosmer-Lemeshow test. Results demonstrated good model fit (*χ*^2^=5.663, *P*=.69).

It should be emphasized that this study primarily focused on evaluating the real-world clinical effectiveness of an AI-enhanced PEWS system rather than on developing or optimizing the underlying model itself. Deployed as a decision support tool, the system provided nurses with clear, objective, and data-driven assistance for triage decisions and interventions, while preserving human clinicians’ ultimate authority to override recommendations—particularly to account for potential transient anomalies in vital signs data.

### Statistical Analysis

SPSS software (version 27.0; IBM Corp) was used to analyze the data. Quantitative data conforming to normal distribution were presented as mean (SD), with intergroup comparisons conducted using the independent sample 2-tailed *t* test. For nonnormally distributed quantitative data, median (IQR) was used, and intergroup comparisons were performed with the Mann-Whitney *U* test. Categorical data were described as frequency (%), with intergroup comparisons analyzed using the chi-square test. Logistic regression was used to evaluate outcome predictors, while ROC curves and calibration curves were used to assess model predictive power and goodness of fit. The significance level was set at α=.05, and *P*<.05 was considered to be statistically significant.

## Results

### PEWS and Emergency Classification and Transfer to the ICU

The mean PEWS of the early warning group was 2.44 (SD 1.41). Of the 597 cases, 68 (11.4%) cases were transferred to the pediatric ICU; the mean PEWS of this group was 3.32 (SD 1.73), including 43 (7.2%) cases of respiratory diseases, 16 (2.7%) cases of nervous system diseases, and 9 (1.5%) cases of circulatory system diseases.

In the early warning group (n=597), there were 23 (3.9%) cases in the first level, 162 (27.1%) cases in the second level, and 412 (69%) cases in the third level. In the non–early warning group (n=636), there were 14 (2.2%) cases in the second level, 619 (97.3%) cases in the third level, and 3 (0.47%) cases in the fourth level.

### Comparison of Hospitalization Duration and Costs Between the 2 Groups

Compared with the non–early warning group, the early warning group showed significantly longer hospitalization duration (*z*=−5.180; *P*<.001) and higher costs (*z*=−6.500; *P*<.001), with statistically significant differences (*P*<.001) between the groups (Table 4).

**Figure 1.**
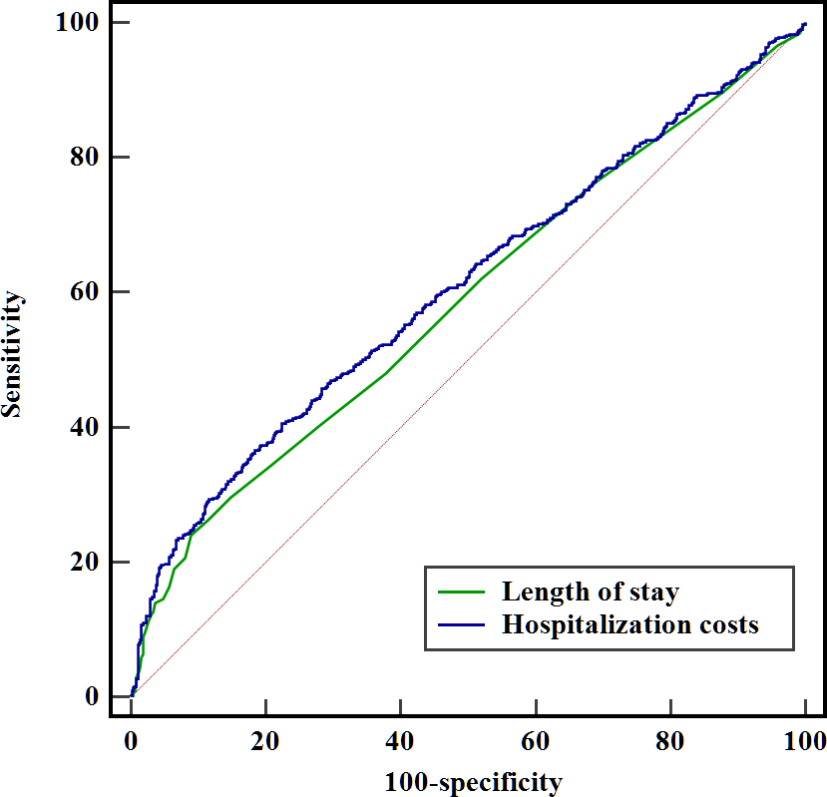
Receiver operating characteristic curves for hospitalization costs and length of stay.

As shown in Table 5, logistic regression analysis revealed that both length of hospital stay and hospitalization costs were independent predictors of study outcomes (all *P*<.001). Specifically, the β coefficient for length of hospital stay was 0.053 (SE 0.010; Wald *χ*^2_1_^=5.5; odds ratio 1.055, 95% CI 1.035‐1.075); and the β coefficient for hospitalization costs was 0.001 (SE 0.000; Wald *χ*^2_1_^=6.1; odds ratio 1.001, 95% CI 1.001‐1.001). These results indicate that increased length of hospital stay and higher hospitalization costs are associated with elevated risks of study outcomes (Figure 1).

**Figure 2.**
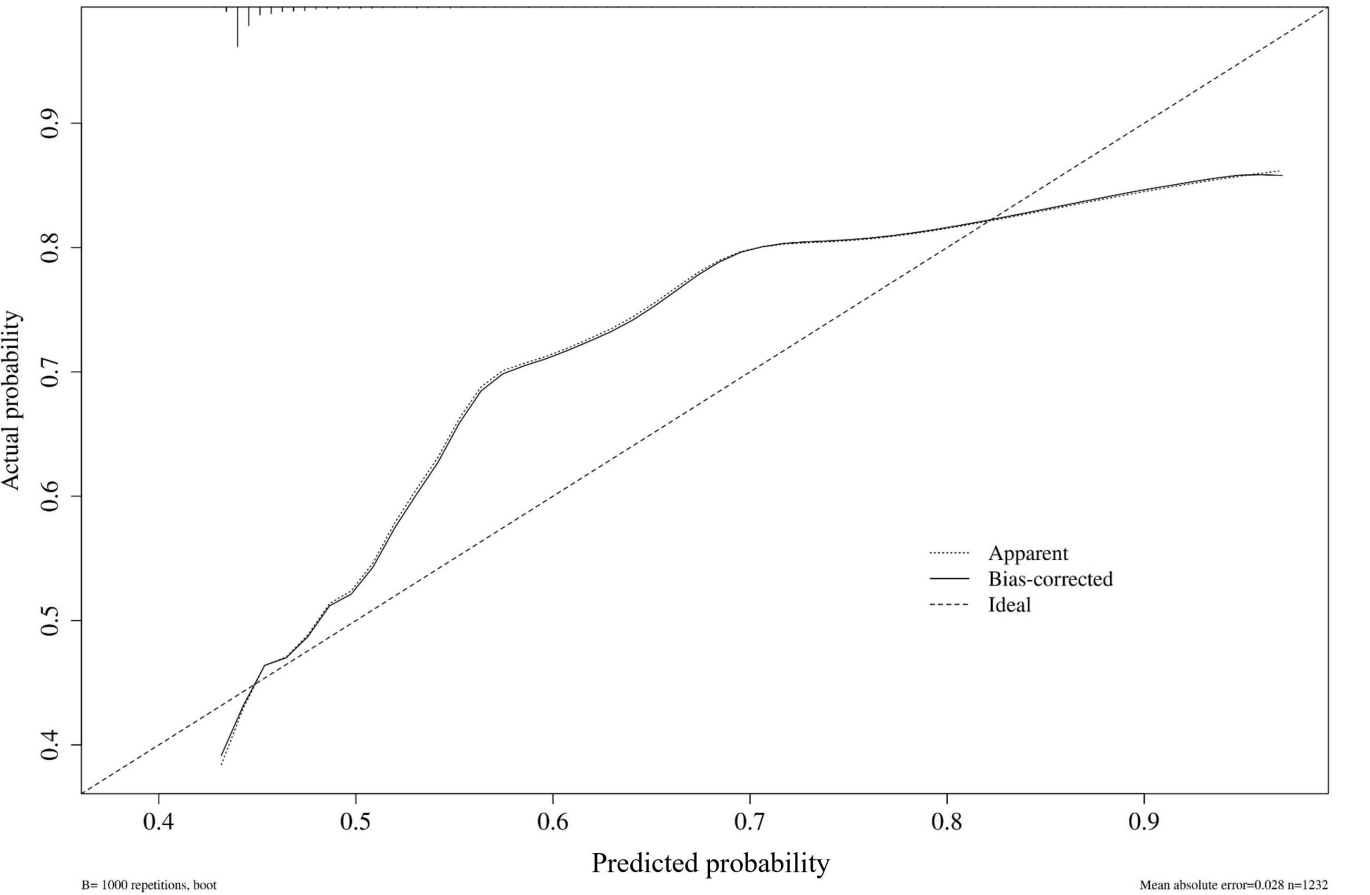
Calibration curves for apparent, bias-corrected, and ideal models.

As shown in Table 6, ROC curve analysis revealed that the area under the curve (AUC) for predicting study outcomes based on hospitalization duration was 0.585 (95% CI 0.553‐0.616), with an accuracy of 0.586, sensitivity of 0.240, specificity of 0.910, positive predictive value of 0.561, and negative predictive value of 0.715. For predicting study outcomes based on hospitalization costs, the AUC was 0.607 (95% CI: 0.575‐0.639), with an accuracy of 0.598, sensitivity of 0.365, specificity of 0.816, positive predictive value of 0.578, and negative predictive value of 0.651. Both AUC values fell within the range of 0.5 to 0.7, indicating certain predictive efficacy for study outcomes. The predictive efficacy of hospitalization costs was slightly superior to that of hospitalization duration (Figure 2).

The calibration curve results demonstrated that both the actual model curve and the bias-corrected curve closely matched the ideal curve, indicating good consistency between the model’s predicted probabilities and the actual incidence rates of outcomes. The Hosmer-Lemeshow test yielded a *χ*^2^=5.7 with a *P=*.69, further confirming the model’s excellent fit and absence of significant calibration bias.

**Table 4.** Comparison of length of hospital stay and hospitalization costs between the early warning group and the non–early warning group.

	Early warning group (n=597)	Non–early warning group (n=636)	*z* score	*P* value
Length of stay (days), median (IQR)	6 (5-11)	6 (4-8)	−5.180	<.001
Hospitalization costs (CNY 1=US $0.146), median (IQR)	4911.79 (2301.05-15,463.47)	3295.17 (1993.88-6884.35)	−6.500	<.001

**Table 5.** Logistic regression results for hospitalization-related variables‌.

Variables	β (SE)		Wald *χ*^2^ (*df*)	Odds ratio (95% CI)	*P* value
Length of stay	0.053 (0.010)		5.533 (1)	1.055 (1.035-1.075)	<.001
Hospitalization costs	0.001 (0.000)		6.075 (1)	1.001 (1.001-1.001)	<.001

**Table 6.** Predictive performance of hospitalization-related variables.

Variables	AUC^a^ (95% CI)	Accuracy	Sensitivity	Specificity	PPV^b^	NPV^c^
Length of stay	0.585 (0.553‐0.616)	0.586	0.240	0.910	0.561	0.715
Hospitalization costs	0.607 (0.575‐0.639)	0.598	0.365	0.816	0.578	0.651

a AUC: area under the curve.

b PPV: positive predictive value.

c NPV: negative predictive value.

### Mortality in the 2 Groups

Among the 597 children in the early warning group, 5 (0.8%) died, including 3 with neurological diseases and 2 with respiratory diseases. No deaths occurred in the non–early warning group.

### Comparison of Length of Stay and Hospitalization Costs by Disease Category Between the 2 Groups

Compared with the non–early warning group, children in the early warning group with respiratory, neurological, or hematologic diseases had significantly longer hospital stays and higher hospitalization costs. The between-group differences were statistically significant (all *P*<.01), as shown in Table 7.

**Table 7.** Comparison of length of hospital stay and hospitalization costs between the early warning group and the non–early warning group by disease category.

Disease category and group	Length of stay (days), median (IQR)	Hospitalization costs (CNY), median (IQR)
Respiratory^a^		
Early warning group (n=463)	6 (4-9)	3770.19 (2092.25-8242.54)
Non–early warning group (n=481)	5 (4-8)	2894.25 (1886.72-5129.65)
Neurological^b^		
Early warning group (n=75)	10 (5-17)	20,603.33 (10,404.37-35,742.30)
Non–early warning group (n=90)	6 (5-11)	6915.65 (2644.04-17,225.70)
Hematologic^c^		
Early warning group (n=59)	20 (6-30)	42,668.25 (8276.94-87,117.50)
Non–early warning group (n=65)	5 (4-10)	5639.23 (2515.09-11,294.13)

a Respiratory: Mann-Whitney U test z score=−3.439 (*P*<.001) for length of stay and z score=−4.334 (*P*<.001) for hospitalization costs.

b Neurological: Mann-Whitney U test z score=−2.661 (*P*=.01) for length of stay and z score=−4.807 (*P*<.001) for hospitalization costs.

c Hematologic: Mann-Whitney U test z score=−4.219 (*P*<.001) for length of stay and z score=−5.291 (*P*<.001) for hospitalization costs.

## Discussion

### Principal Findings

Huang et al [18] suggested that hospitals should develop an information system for early warning of children’s conditions, providing guidance and clinical decision support for nurses. This would help nurses accurately identify and manage the risk of deterioration in children’s condition at an early stage, thereby improving patient treatment outcomes. As medical and nursing care models evolve, using information systems to strengthen the early recognition of clinical deterioration in children—guiding nurses to implement appropriate interventions—is increasingly important for improving the specificity and effectiveness of nursing care. In this study, the information system was used to generate PEWS-related warning prompts and color-coded alerts, and nurses managed patients accordingly based on the score (Multimedia Appendix 1 shows screenshots of the PEWS color-coded alerts). This approach aligns with the suggestion by Xuefen et al [19] that future studies may apply the PEWS to predict and assess disease severity in other pediatric conditions and develop an information-based PEWS system to enable automated early warnings.

Our results showed that, compared with the non–early warning group, children in the early warning group had a significantly longer length of hospital stay and significantly higher hospitalization costs (*P*<.001). However, this association likely reflects a severity spectrum: patients with greater physiological instability naturally had a higher PEWS and concurrently needed more intensive resource investment. The modest discriminative ability of resource metrics for PEWS status (AUC 0.585‐0.607) showed the substantial overlap between groups, supporting that PEWS activation identifies a broad phenotype of physiological disturbance rather than a specific high-risk subgroup with unique resource consumption patterns. From an operational point of view, this association is still valuable: early identification of patients who are likely to consume substantial resources (median cost difference: CN ¥1617 [US $238]; median length of stay difference: 1 day) may inform capacity planning and nursing workload allocation, even if the relationship is mediated through illness severity. These results suggest that using an AI system was meaningful for establishing an objective, accurate, rapid, and easily implementable PEWS system in pediatric emergency care. It helped pediatric emergency nurses use objective assessment indicators to promptly identify abnormal physiological signs, rapidly and accurately recognize critically ill children, and quickly differentiate those who should be prioritized in nursing care (higher-acuity emergency) and those who can safely wait (lower-acuity emergency). This supports targeted medical and nursing interventions [20], reduces the limitations of experience-based judgment, minimizes errors arising from subjective assessment, improves overall treatment effectiveness, and enhances caregivers’ satisfaction [4,21]. A reliable pediatric triage tool is crucial for nurses working in the pediatric emergency department [22,23]. The findings also indicate that children in the early warning group require closer observation after admission. In addition to improving the targeting of nursing care, an information system–based PEWS may reduce nurses’ workload and improve efficiency. Clinical decision support systems have been shown to enhance nursing efficiency [24,25].

Furthermore, compared with the non–early warning group, children in the PEWS early warning group within the top 3 admission categories in pediatric emergency care—respiratory, neurological, and hematologic diseases—had longer hospital stays and higher hospitalization costs (*P*<.001). This suggests that patients with these conditions who triggered PEWS activation via the information system were more severely ill, which may explain the longer hospitalization and higher costs. When stratified by disease category, the resource disparity was most pronounced in hematologic disorders (median length of stay 20 vs 5 days; costs CN ¥42,668 [US $6274] vs CN ¥5639 [US $829]), followed by neurological and respiratory diseases. These patterns suggest that the activation of AI-based PEWS triggers within specific diagnostic categories may alert clinicians to patients with complicated disease courses who need to be observed and managed for a longer period. Zixian and Jin [26] reported that the optimal PEWS trigger values for predicting ICU transfer may differ across specialties, and there is currently no unified clinical standard.

PEWS is important for assessing disease severity in children. In this study, of the 597 children in the early warning group, 68 (11.4%) were transferred to the pediatric ICU, with a mean PEWS of 3.32 (SD 1.73), which was higher than the score among children transferred to general wards. No children in the non–early warning group were transferred to the pediatric ICU. The proportions of emergency triage level 1 and level 2 cases were higher in the early warning group than in the non–early warning group, and no level 1 cases were observed in the non–early warning group. In addition, there were 5 (0.8%) deaths in the early warning group (3 neurological and 2 respiratory), whereas no deaths occurred in the non–early warning group (n=636). Furthermore, 619 (97.3%) children in the non–early warning group were triaged as level 3. These findings indicate that, in pediatric emergency care, a higher PEWS activated via the information system is associated with greater disease severity. This is consistent with the view of Özel et al [27] that PEWS is a strong determinant of mortality in critically injured pediatric trauma patients. While this concentration of adverse events within the triggered group suggests that PEWS captures clinically relevant physiological disorders, the absence of events in the nontriggered group reflects a spectrum bias inherent to our cohort (predominantly lower-acuity presentations) rather than proving the score’s negative predictive value. The retrospective design precludes determination of whether PEWS provided early warning (preceding deterioration) or merely concurrent documentation of established illness severity. Huang et al [18] reported that nurses’ understanding of PEWS may be incomplete, underscoring the need for systematic training to improve nurses’ awareness and capability in early recognition of clinical deterioration in children.

The value of this analysis is in the characterization of the resource burden that is associated with the activation of the AI-PEWS when the system is implemented. For nursing management, the association between the alerts and the following length of stay and costs can provide empirical support for dynamic staffing models. We note that one should not use these results to conclude that PEWS itself improves outcomes or that patients who do not trigger a warning are at low risk for adverse events. The design of the study does not allow for the conclusion of causality.

### Limitations

As a single-center retrospective study, this work is subject to confounding by indication, which is inherent in observational research. Specifically, physiologically unstable patients are more likely to trigger a higher PEWS while simultaneously requiring more intensive resource use; therefore, we cannot make causal inferences regarding the independent predictive value of the AI-based PEWS. Also, the modest discriminative performance (AUC 0.585‐0.607) and the absence of hard clinical end points limit the ability to claim prognostic utility for clinical deterioration. Furthermore, as an early implementation-phase study, the AI-based PEWS functioned only as an auxiliary decision support tool for clinical judgment rather than as an autonomous predictor. These results characterize baseline operational patterns rather than definitive risk stratification, and prospective multicenter validation is needed. The limitations of using AI for early warning include sensitivity to outliers and noise; for example, vital sign data may exhibit instantaneous abnormalities due to patient restlessness. After the AI system triggers an alert or suggests intervention measures, clinicians should comprehensively consider and use AI as an auxiliary tool.

### Conclusions

In summary, compared with the non–early warning group, the early warning group had significantly longer hospital stays and higher hospitalization costs; similar patterns were observed within respiratory, neurological, and hematologic disease categories. The study showed differences between children who triggered the warning and children who did not, providing a reference for identifying critically ill children and for targeted care. This study reports on the association between early warning alerts and subsequent resource use and provides an empirical baseline for nursing workload allocation and emergency department capacity management. We stress that these observational data reflect underlying disease severity (subject to confounding by indication) and do not show that the AI-based PEWS has independent predictive validity for resource use, which would be expected from the underlying clinical condition. Therefore, while these results can be useful for planning and organizing targeted care, they should not be interpreted as meaning that the AI-based PEWS caused or independently predicted resource use.

## Supplementary material

10.2196/89306 Multimedia Appendix 1 Screenshots of the Pediatric Early Warning Score color-based alerts.

## Abbreviations

AI: artificial intelligence
AUC: area under the curve
ICU: intensive care unit
PEWS: Pediatric Early Warning Score
ROC: receiver operating characteristic
