# Supplementary figures and images for: Application of an AI-Based Pediatric Early Warning Score in the Pediatric Emergency Department: Cross-Sectional Study
Source: JMIR Form Res. 2026 May 19;10:e89306. doi: 10.2196/89306 (PMC13186435; doi:10.2196/89306)

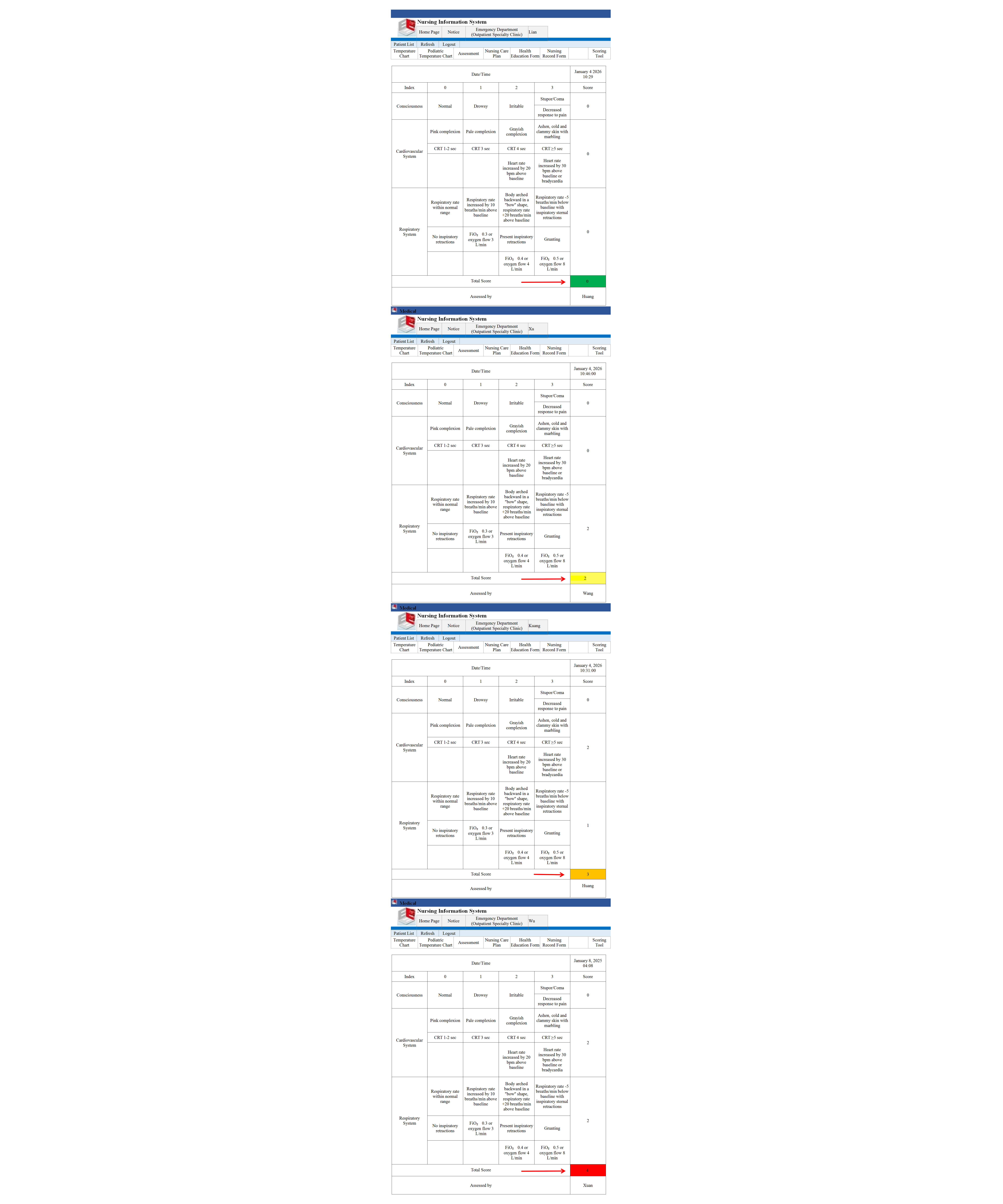

Supplement: Multimedia Appendix 1 [file formative-v10-e89306-s001.png]
